# Age-related risk factors for leftover drugs: a lifespan analysis in community pharmacies

**DOI:** 10.3389/jpps.2026.16879

**Published:** 2026-09-04

**Authors:** Toshiyuki Hirai, Shunsuke Hanaoka, Yuusuke Terakado, Toshiichi Seki, Hiroyuki Hayashi, Fumiyuki Watanabe

**Affiliations:** 1 Department of Pharmacy, Hitachi, Ltd. Hitachinaka General Hospital, Ibaraki, Japan; 2 Laboratory of Pharmacotherapy, School of Pharmacy, Nihon University, Chiba, Japan; 3 Laboratory of Pharmacy Practice in Primary Care, School of Pharmacy, Nihon University, Chiba, Japan

**Keywords:** age-specific factors, community pharmacy, leftover drugs, medication adherence, polypharmacy

## Abstract

**Introduction:**

Medication adherence is a critical global challenge because suboptimal adherence reduces therapeutic efficacy and increases healthcare costs. While leftover drugs are vital indicators of potential adherence-related challenges, age-specific factors contributing to their occurrence, particularly across the lifespan, remain insufficiently understood. This study was aimed at clarifying age-stratified factors associated with leftover drugs using large-scale objective data from protocol-based pharmacotherapy management (PBPM) in community pharmacies.

**Methods:**

This was a single-center, retrospective, observational cohort study of 36,340 outpatients. Objective reporting of data based on the PBPM in community pharmacies between 2016 and 2021 was analyzed. Multivariate logistic regression and predicted probability models were used to evaluate the interactions between age (decade-stratified) and four primary clinical factors: drug count, prescription duration, prescribing department count, and sex.

**Results:**

The overall proportion of leftover drugs was 9.6% (*n* = 3,472). In the overall population comparison, no significant differences were observed in terms of sex, but the patients in the leftover group were significantly older and had higher medication counts, longer prescription durations, and more prescribing departments. Multivariate analysis revealed significant interactions between age and each of the three primary prescription-related factors. The impact of clinical characteristics on the proportion of leftover drugs varied distinctly across life stages. Specifically, long-term prescription was the most potent risk factor for younger adults (20s–40s), whereas polypharmacy and multidepartment visits were the primary factors associated with leftover drugs for older patients. Predicted probability models indicated that having fewer than five prescribed medications was consistently associated with a lower likelihood of leftover drugs across all age groups.

**Conclusion:**

This study highlights the influence of clinical characteristics on leftover drug shifting substantially with age. Consequently, effective management requires age-specific interventions centered on minimizing drug counts, with an emphasis on optimizing prescription duration in younger adults and promoting medication simplification and inter-institutional coordination in older adults.

## Introduction

The World Health Organization estimates medication adherence among patients with chronic diseases in developed countries at approximately 50%. Reduced adherence diminishes therapeutic effects, worsens the quality of life, and imposes a significant burden on medical economics by increasing unnecessary healthcare costs, making it a prominent international challenge [1]. In Japan, the total value of leftover drugs, defined as potential doses missed by patients receiving home medical care, is estimated at approximately JPY 50 billion annually. This situation raises concerns regarding the wastage of medical resources and increased health risks associated with improper medication use [2]. Against this background, the Japan’s medical fee system now stipulates that pharmacies must confirm the presence of leftover drugs and implement necessary prescription adjustments [3–5].

The presence of leftover drugs is a critical indicator of potential adherence-related challenges and provides essential information for understanding the treatment status of patients [6]. Therefore, elucidating the underlying factors of leftover drug occurrence and contributing to the optimization of prescriptions are directly linked to improving adherence and realizing safe drug therapy.

Collaborative drug therapy management, a new form of pharmacotherapy management in which pharmacists intervene following protocols based on contracts with physicians, was introduced in the United States [7]. In Japan, a similar initiative known as protocol-based pharmacotherapy management (PBPM) is increasingly becoming prevalent. In PBPM, pharmacists collaborate with physicians to administer drug therapy based on pre-approved protocols [8]. At our medical institution, we introduced and have been operating a standardized “leftover drug adjustment protocol” since April 2016. This protocol allows community pharmacists to adjust prescription duration based on their objective assessment of the patient’s leftover drug status, typically verified by physical counts [9].

For pharmacists to intervene effectively against leftover drugs in clinical settings, an understanding of which patient demographics stratified by age group are prone to generating leftover drugs is extremely important. However, to date, pharmacist intervention records under PBPM have not been utilized for performing a detailed comparison of leftover drug occurrence by decade or for clarifying the relationship between age groups and causative factors.

In this study, we used reporting data from leftover drug adjustments based on PBPM operations in community pharmacies to classify outpatients into decade-stratified age groups and to investigate the association between the presence of leftover drugs and prescription-related factors. This study was aimed at identifying the specific factors associated with the occurrence of leftover drugs at each life stage and propose optimal countermeasures and medication support strategies tailored to age-specific characteristics.

## Materials and methods

### Study design and ethics

This single-center, retrospective, observational cohort study was conducted in accordance with the Declaration of Helsinki and current ethical guidelines. The study design and protocol, including the opt-out method for obtaining consent, were approved by the ethics committee of Hitachi Ltd. Hitachinaka General Hospital (approval no: BOE-38-001_202502-04). As an observational study using existing electronic medical record (EMR) data, no direct patient intervention was performed.

### Study population

**FIGURE 1:**
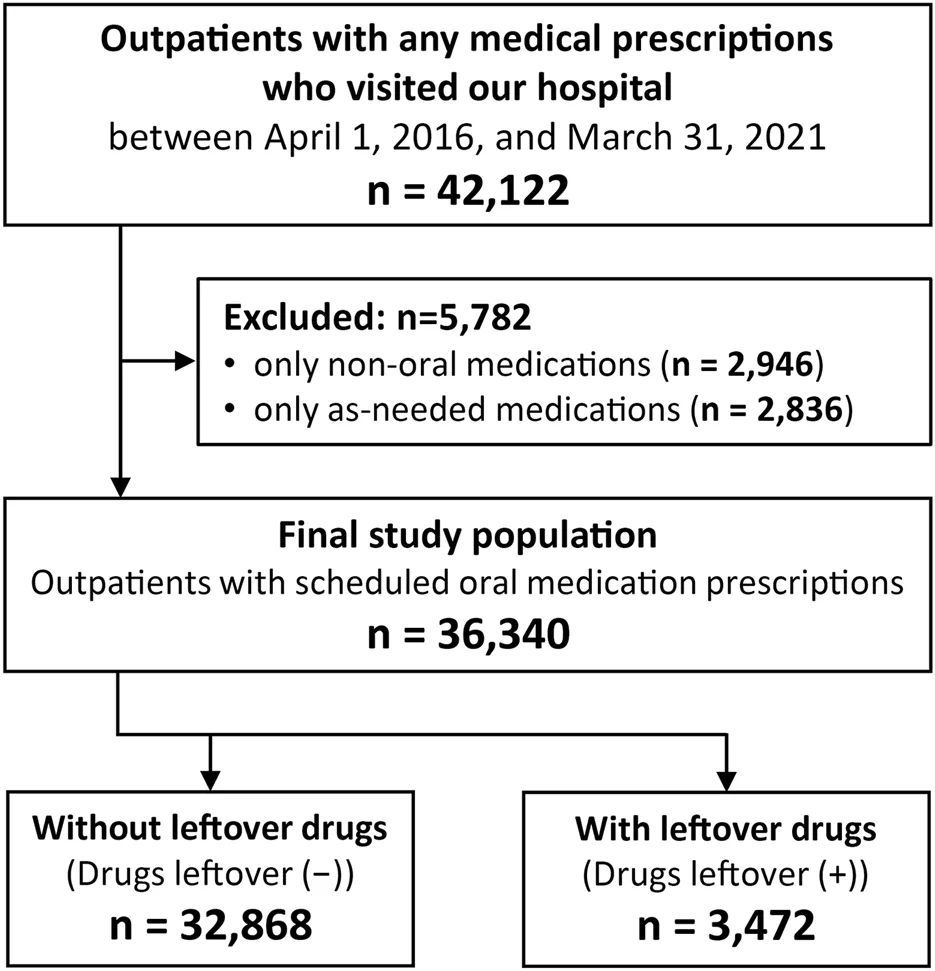
Flowchart of the patient selection process. This diagram illustrates the inclusion and exclusion criteria for the study population. A total of 36,340 outpatients were analyzed, categorized into the leftover drug group and the non-leftover drug group based on reporting from protocol-based pharmacotherapy management.

We initially identified outpatients who visited Hitachi Ltd. Hitachinaka General Hospital and received medical prescriptions between April 1, 2016, and March 31, 2021. We focused on patients requiring ongoing medication management to ensure consistent evaluation of medication adherence. Therefore, patients were excluded if they received only non-oral (e.g., injectables or topical agents) or only as-needed (pro re nata) medications. Consequently, the final study population comprised outpatients scheduled for oral medication prescriptions. Based on the leftover drug adjustment protocol, patients for whom leftover drugs were reported were categorized into the leftover drug group (Drugs leftover (+)), whereas those with no such reports were categorized into the non-leftover drug group (Drugs leftover (−)). The patient selection process is illustrated in Figure 1.

### Identification of leftover drugs

Leftover drugs were managed and reported according to a standardized leftover drug adjustment protocol. Briefly, this protocol enabled community pharmacists to identify leftover drugs through patient interviews and physical counselling during the dispensing process. This was followed by providing objective reports to the prescribing physician to facilitate subsequent adjustments in prescription duration [9]. These objective reports served as the primary basis for identifying patients in the “with leftover drugs” group (Drugs leftover (+)). Specifically, patients who had at least one report (≥1 occurrence) of leftover drugs during the study period were classified into the Drugs leftover (+) group, whereas those with strictly zero reports were classified into the Drugs leftover (−) group.

### Variables

Patient information, including sex, age, drug counts, drug prescription days, and prescription department counts, was retrospectively retrieved from the EMRs.

Age was defined as the age at first visit during the study period. To clarify age-related trends, participants were categorized into ten age groups based on 10-year increments: <10 years, 10s (10–19 years), 20s (20–29 years), 30s (30–39 years), 40s (40–49 years), 50s (50–59 years), 60s (60–69 years), 70s (70–79 years), 80s (80–89 years), and ≥90 years.

Drug counts were defined as the number of oral medications prescribed at our outpatient clinic. Based on prior research on polypharmacy [10], patients were categorized as having “<5 drugs” or “≥5 drugs.”

Drug prescription days was the maximum number of days per prescription during the study period. Based on previous studies [9], these were categorized as “<30 days,” “30–59 days,” or “≥60 days.”

Prescribing departments’ counts were the total number of departments visited during the study period (out of 24 departments available at the hospital). Considering the data distribution and ease of clinical interpretation, patients were categorized into three groups: “1 department,” “2 departments,” or “≥3 departments.”

### Statistical analysis

A series of analyses using logistic regression was performed to determine whether factors associated with leftover drugs varied by age group. All statistical analyses were conducted using JMP Student Edition 19 (SAS Institute Inc., Cary, NC, USA).

#### Preliminary analysis

**FIGURE 2:**
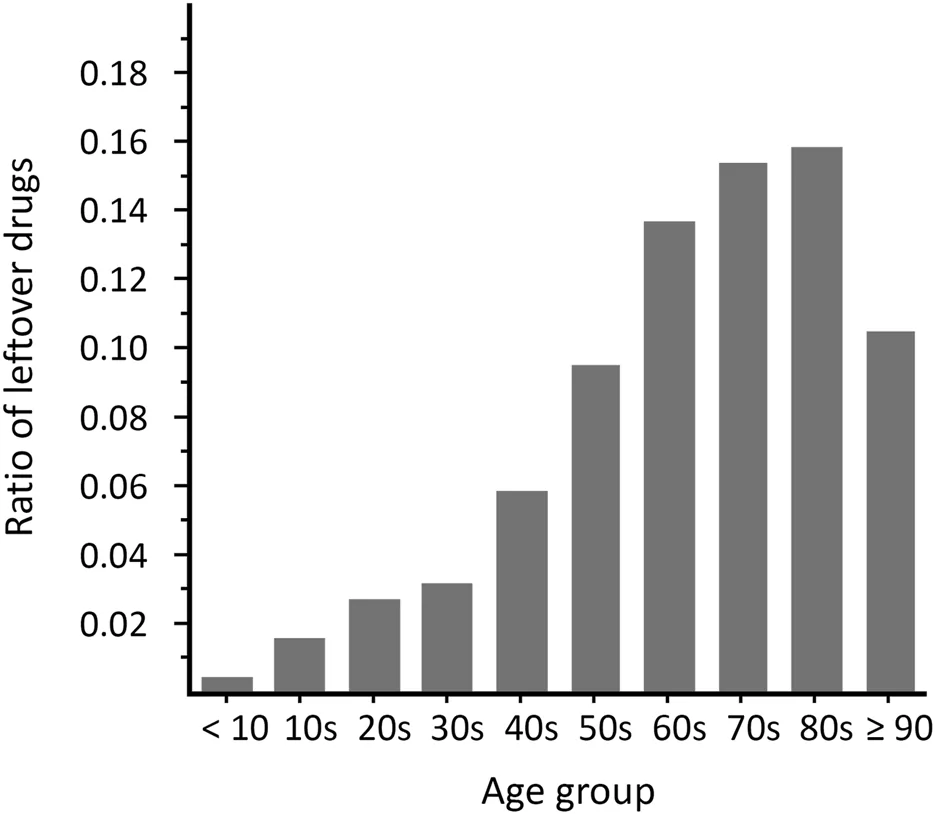
Prevalence of leftover drugs across decade-stratified age groups. The bar graph displays the percentage of patients taking leftover drugs in each age group. A specific trend was observed across the lifespan, including a decrease in prevalence among the oldest old (aged 90 years and older).

First, basic characteristics and clinical factors were compared between the Drugs leftover (+) and Drugs leftover (−) groups for the entire population (Table 1). Age-stratified subgroup comparisons were conducted to identify exploratory trends (Table 2). As part of this preliminary analysis, the actual (observed) ratio of leftover drugs for each age group was calculated, and the resulting trends are illustrated in a graph (Figure 2). Continuous variables were expressed as mean and standard deviation (SD) and compared using Welch’s *t*-test, not requiring the assumption of equal variance. Categorical variables (such as sex) were expressed as numbers and percentages (%) and compared using the *χ* ^*2*^ test. The variables included in the analysis were sex, age, prescription department count, drug count, and drug prescription days. Additionally, within the Drugs leftover (+) group, the frequency distribution of leftover drug reports (1, 2, or ≥3 occurrences) across age groups was compared using the *χ* ^*2*^ test (Supplementary Table S1).

**TABLE 1:** Comparison of basic characteristics and clinical factors between patients with and without leftover drugs in the overall population.

Variables	Overall (n or Mean)	Overall (Ratio (%) or SD)	Drugs leftover (−) (n or Mean)	Drugs leftover (−) (Ratio (%) or SD)	Drugs leftover (+) (n or Mean)	Drugs leftover (+) (Ratio (%) or SD)	*p-value*
Sex	​	​	​	​	​	​	0.261
Female	16,887	46.5%	15,305	46.6%	1,582	45.6%	​
Male	19,453	53.5%	17,563	53.4%	1890	54.4%	​
Age (years)	52.9	25.8	51.3	26.1	67.9	15.4	<0.0001
<10 years	3,325	9.2%	3,311	10.1%	14	0.4%	<0.0001
10s	2,325	6.4%	2,289	7.0%	36	1.0%	​
20s	2,159	5.9%	2,101	6.4%	58	1.7%	​
30s	2,725	7.5%	2,639	8.0%	86	2.5%	​
40s	3,761	10.3%	3,541	10.8%	220	6.3%	​
50s	3,984	11.0%	3,604	11.0%	380	10.9%	​
60s	5,515	15.2%	4,761	14.5%	754	21.7%	​
70s	7,613	20.9%	6,439	19.6%	1,174	33.8%	​
80s	4,324	11.9%	3,638	11.1%	686	19.8%	​
≥90 years	609	1.7%	545	1.7%	64	1.8%	​
Drug counts	2.5	2.0	2.3	1.8	4.5	2.6	<0.0001
<5 drugs	31,436	86.5%	29,434	89.6%	2002	57.7%	<0.0001
≥5 drugs	4,904	13.5%	3,434	10.4%	1,470	42.3%	​
Drug prescription days	41.7	36.6	38.1	35.9	75.6	23.5	<0.0001
<30 days	17,972	49.5%	17,825	54.2%	147	4.2%	<0.0001
30–59 days	5,251	14.5%	4,730	14.4%	521	15%	​
≥60 days	13,115	36.1%	10,311	31.4%	2,804	80.8%	​
Prescribing departments counts	1.5	0.9	1.4	0.8	2.1	1.3	<0.0001
1 department	25,487	70.1%	24,057	73.2%	1,430	41.2%	<0.0001
2 departments	7,030	19.3%	6,025	18.3%	1,005	28.9%	​
≥3 departments	3,823	10.5%	2,786	8.5%	1,037	29.9%	​

A total of 36,340 patients were categorized into the non-leftover drug group (Drugs leftover (−)) and the leftover drug group (Drugs leftover (+)). Comparisons were made for sex, age, drug count, drug prescription days, and prescribing department count. Continuous variables are expressed as mean ± standard deviation (SD), and categorical variables are presented as numbers (*n*) and percentages (%).

**TABLE 2:** Age-stratified comparison of patient characteristics between groups with and without leftover drugs.

<10 years (*n* = 3,325)	Drugs leftover (−)		Drugs leftover (+)		*p*-value
	*n* = 3,311 (99.6%)		*n* = 14 (0.4%)		
Variables	*n* or mean	Ratio (%) or SD	*n* or mean	Ratio (%) or SD	
Sex	​	​	​	​	0.587
Female	1,421	42.9%	5	35.7%	​
Male	1890	57.1%	9	64.3%	​
Drug counts	2.0	1.0	2.4	1.2	0.263
<5 drugs	3,075	92.9%	7	50%	<0.0001
≥5 drugs	236	7.1%	7	50%	​
Drug prescription days	18.6	24.7	65.9	19.3	<0.0001
<30 days	2,607	78.8%	1	7.1%	<0.0001
30–59 days	298	9.0%	1	7.1%	​
≥60 days	405	12.2%	12	85.7%	​
Prescribing departments counts	1.1	0.4	1.5	0.7	0.041
1 department	3,014	91.0%	8	57.1%	<0.0001
2 departments	259	7.8%	5	35.7%	​
≥3 departments	38	1.1%	1	7.1%	​

10s (n = 2,325)	Drugs leftover (−)		Drugs leftover (+)		*p*-value
	*n* = 2,289 (98.5%)		*n* = 36 (1.5%)		
Variables	*n* or mean	Ratio (%) or SD	*n* or mean	Ratio (%) or SD	
Sex	​	​	​	​	0.383
Female	1,041	45.5%	19	52.8%	​
Male	1,248	54.5%	17	47.2%	​
Drug counts	1.8	0.9	2.6	1.7	0.005
<5 drugs	2,176	95.1%	30	83.3%	0.002
≥5 drugs	113	4.9%	6	16.7%	​
Drug prescription days	34.3	33.8	69.7	18.3	<0.0001
<30 days	1,276	55.7%	1	2.8%	<0.0001
30–59 days	349	15.2%	6	16.7%	​
≥60 days	664	29.0%	29	80.6%	​
Prescribing departments counts	1.2	0.5	1.8	1.3	0.005
1 department	1947	85.1%	22	61.1%	<0.0001
2 departments	288	12.6%	6	16.7%	​
≥3 departments	54	2.4%	8	22.2%	​

20s (n = 2,159)	Drugs leftover (−)		Drugs leftover (+)		*p*-value
	*n* = 2,101 (97.3%)		*n* = 58 (2.7%)		
Variables	*n* or mean	Ratio (%) or SD	*n* or mean	Ratio (%) or SD	
Sex	​	​	​	​	0.646
Female	1,095	52.1%	32	55.2%	​
Male	1,006	47.9%	26	44.8%	​
Drug counts	1.9	1.1	3.8	2.2	<0.0001
<5 drugs	2034	96.8%	37	63.8%	<0.0001
≥5 drugs	67	3.2%	21	36.2%	​
Drug prescription days	19.8	28.4	76.3	22.5	<0.0001
<30 days	1,689	80.4%	2	3.4%	<0.0001
30–59 days	152	7.2%	8	13.8%	​
≥60 days	260	12.4%	48	82.8%	​
Prescribing departments counts	1.2	0.6	1.8	1.5	0.002
1 department	1776	84.5%	36	62.1%	<0.0001
2 departments	247	11.8%	9	15.5%	​
≥3 departments	78	3.7%	13	22.4%	​

30s (n = 2,725)	Drugs leftover (−)		Drugs leftover (+)		*p*-value
	*n* = 2,639 (96.8%)		*n* = 86 (3.2%)		
Variables	*n* or mean	Ratio (%) or SD	*n* or mean	Ratio (%) or SD	
Sex	​	​	​	​	0.331
Female	1,394	52.8%	50	58.1%	​
Male	1,245	47.2%	36	41.9%	​
Drug counts	2.0	1.2	4.2	2.9	<0.0001
<5 drugs	2,536	96.1%	53	61.6%	<0.0001
≥5 drugs	103	3.9%	33	38.4%	​
Drug prescription days	22.9	29.3	76.5	22.0	<0.0001
<30 days	1991	75.4%	3	3.5%	<0.0001
30–59 days	251	9.5%	13	15.1%	​
≥60 days	397	15.0%	70	81.4%	​
Prescribing departments counts	1.3	0.7	1.7	1.1	0.001
1 department	2,127	80.6%	47	54.7%	<0.0001
2 departments	361	13.7%	25	29.1%	​
≥3 departments	151	5.7%	14	16.3%	​

40s (n = 3,761)	Drugs leftover (−)		Drugs leftover (+)		*p*-value
	*n* = 3,541 (94.2%)		*n* = 220 (5.9%)		
Variables	*n* or mean	Ratio (%) or SD	*n* or mean	Ratio (%) or SD	
Sex	​	​	​	​	0.061
Female	1,653	46.7%	117	53.2%	​
Male	1888	53.3%	103	46.8%	​
Drug counts	2.0	1.4	3.9	2.5	<0.0001
<5 drugs	3,337	94.2%	142	64.5%	<0.0001
≥5 drugs	204	5.8%	78	35.5%	​
Drug prescription days	31.8	33.7	72.8	21.9	<0.0001
<30 days	2,221	62.7%	6	2.7%	<0.0001
30–59 days	492	13.9%	39	17.7%	​
≥60 days	828	23.4%	175	79.5%	​
Prescribing departments counts	1.3	0.8	1.7	1.2	<0.0001
1 department	2,724	76.9%	129	58.6%	<0.0001
2 departments	585	16.5%	54	24.5%	​
≥3 departments	232	6.6%	37	16.8%	​

50s (n = 3,984)	Drugs leftover (−)		Drugs leftover (+)		*p*-value
	*n* = 3,604 (90.5%)		*n* = 380 (9.5%)		
Variables	*n* or mean	Ratio (%) or SD	*n* or mean	Ratio (%) or SD	
Sex	​	​	​	​	0.144
Female	1,632	45.3%	187	49.2%	​
Male	1972	54.7%	193	50.8%	​
Drug counts	2.2	1.8	4.4	2.7	<0.0001
<5 drugs	3,283	91.1%	233	61.3%	<0.0001
≥5 drugs	321	8.9%	147	38.7%	​
Drug prescription days	37.7	34.5	73.0	21.2	<0.0001
<30 days	1973	54.7%	18	4.7%	<0.0001
30–59 days	510	14.2%	74	19.5%	​
≥60 days	1,121	31.1%	288	75.8%	​
Prescribing departments counts	1.4	0.8	1.8	1.1	<0.0001
1 department	2,651	73.6%	199	52.4%	<0.0001
2 departments	661	18.3%	103	27.1%	​
≥3 departments	292	8.1%	78	20.5%	​

60s (n = 5,515)	Drugs leftover (−)		Drugs leftover (+)		*p*-value
	n = 4,761 (86.3%)		n = 754 (13.7%)		
Variables	*n* or mean	Ratio (%) or SD	*n* or mean	Ratio (%) or SD	
Sex	​	​	​	​	0.152
Female	2,115	44.4%	356	47.2%	​
Male	2,646	55.6%	398	52.8%	​
Drug counts	2.4	2.0	4.7	2.7	<0.0001
<5 drugs	4,178	87.8%	425	56.4%	<0.0001
≥5 drugs	583	12.2%	329	43.6%	​
Drug prescription days	45.8	37.8	74.0	25.0	<0.0001
<30 days	2,168	45.5%	47	6.2%	<0.0001
30–59 days	749	15.7%	119	15.8%	​
≥60 days	1844	38.7%	588	78.0%	​
Prescribing departments counts	1.4	0.9	2.0	1.3	<0.0001
1 department	3,329	69.9%	346	45.9%	<0.0001
2 departments	995	20.9%	211	28%	​
≥3 departments	437	9.2%	197	26.1%	​

70s (n = 7,613)	Drugs leftover (−)		Drugs leftover (+)		*p*-value
	*n* = 6,439 (84.6%)		*n* = 1,174 (15.4%)		
Variables	*n* or mean	Ratio (%) or SD	*n* or mean	Ratio (%) or SD	
Sex	​	​	​	​	0.030
Female	2,880	44.7%	485	41.3%	​
Male	3,559	55.3%	689	58.7%	​
Drug counts	2.6	2.1	4.4	2.5	<0.0001
<5 drugs	5,499	85.4%	680	57.9%	<0.0001
≥5 drugs	940	14.6%	494	42.1%	​
Drug prescription days	51.6	36.9	76.9	23.2	<0.0001
<30 days	2,379	37.0%	43	3.7%	<0.0001
30–59 days	1,121	17.4%	163	13.9%	​
≥60 days	2,938	45.6%	968	82.5%	​
Prescribing departments counts	1.6	0.9	2.3	1.4	<0.0001
1 department	4,010	62.3%	430	36.6%	<0.0001
2 departments	1,550	24.1%	359	30.6%	​
≥3 departments	879	13.7%	385	32.8%	​

80s (n = 4,324)	Drugs leftover (−)		Drugs leftover (+)		*p*-value
	*n* = 3,638 (84.1%)		*n* = 686 (15.9%)		
Variables	*n* or mean	Ratio (%) or SD	*n* or mean	Ratio (%) or SD	
Sex	​	​	​	​	0.018
Female	1727	47.5%	292	42.6%	​
Male	1911	52.5%	394	57.4%	​
Drug counts	3.0	2.4	4.7	2.5	<0.0001
<5 drugs	2,894	79.5%	370	53.9%	<0.0001
≥5 drugs	744	20.5%	316	46.1%	​
Drug prescription days	51.3	35.0	78.1	24.3	<0.0001
<30 days	1,293	35.5%	24	3.5%	<0.0001
30–59 days	695	19.1%	87	12.7%	​
≥60 days	1,650	45.4%	575	83.8%	​
Prescribing departments counts	1.6	1.0	2.5	1.4	<0.0001
1 department	2,139	58.8%	193	28.1%	<0.0001
2 departments	948	26.1%	214	31.2%	​
≥3 departments	551	15.1%	279	40.7%	​

≥ 90 years (n = 609)	Drugs leftover (−)		Drugs leftover (+)		*p*-value
	*n* = 545 (89.5%)		*n* = 64 (10.5%)		
Variables	*n* or mean	Ratio (%) or SD	*n* or mean	Ratio (%) or SD	
Sex	​	​	​	​	0.668
Female	347	63.7%	39	60.9%	​
Male	198	36.3%	25	39.1%	​
Drug counts	2.9	2.5	5.7	2.8	<0.0001
<5 drugs	422	77.4%	25	39.1%	<0.0001
≥5 drugs	123	22.6%	39	60.9%	​
Drug prescription days	44.5	34.4	75.2	22.8	<0.0001
<30 days	228	41.8%	2	3.1%	<0.0001
30–59 days	113	20.7%	11	17.2%	​
≥60 days	204	37.4%	51	79.7%	​
Prescribing departments counts	1.6	0.9	2.3	1.3	<0.0001
1 department	340	62.4%	20	31.3%	<0.0001
2 departments	131	24.0%	19	29.7%	​
≥3 departments	74	13.6%	25	39.1%	​

Participants were stratified into groups based on 10-year age increments to summarize clinical differences between the Drugs leftover (+) and (−) groups within each generation. Column headers for each age section indicate the total number of patients (*n*) in that group, with individual cells showing the distribution within each age category. Statistical significance for group comparisons was evaluated using the *χ* ^*2*^ test.

#### Multivariate logistic regression model construction

For the ease of clinical interpretation and to accommodate nonlinear relationships, all variables were entered into a multivariate logistic regression model as categorical variables. To construct the model, the following categories were established as a reference: female, age group: <10 years, drug count: <5 drugs, drug prescription days: <30 days, and prescribing department count: 1 department.

#### Global model evaluation and interaction testing

A multivariate logistic regression model was constructed with leftover drug status as the dependent variable to verify whether the effects of clinical factors varied by age group (interaction). The global model included the main effects of all factors as well as all “factor × age group” interaction terms. Model fit was assessed using the *p*-value of the overall likelihood ratio test; explanatory power was evaluated by generalized *R* ^2^; and discriminative ability was measured using the area under the curve (AUC). The influence of each factor on the presence of leftover drugs was quantified using *β* coefficients, adjusted odds ratios (AOR), and 95% confidence intervals (95% CI). The *β* coefficient was defined as *β* = ln (OR). The presence of an interaction was determined based on whether the *p*-value for each interaction term in the effect likelihood ratio test was less than 0.05. The results are presented in Table 3. This global model was used to identify the effects of clinical factors on leftover drugs, moderated significantly by age.

**TABLE 3:** Multivariate logistic regression analysis of factors influencing leftover drug occurrence and their interaction with age group.

*n* = 36,340, *p* < 0.0001, *R* ^2^ = 0.323, AUC = 0.858				
Variables	*β*	AOR	95% CI	*p*-value
Sex				
Male vs. Female	−0.12	0.89	(0.75–1.05)	0.166
Age (years)				
10s vs. <10 years	0.73	2.09	(0.49–8.79)	0.317
20s vs. <10 years	1.65	5.23	(1.39–19.71)	0.015
30s vs. <10 years	1.65	5.23	(1.47–18.60)	0.011
40s vs. <10 years	2.05	7.79	(2.30–26.34)	0.001
50s vs. <10 years	2.68	14.63	(4.44–48.15)	<0.0001
60s vs. <10 years	3.14	23.07	(7.08–75.22)	<0.0001
70s vs. <10 years	2.97	19.50	(5.99–63.50)	<0.0001
80s vs. <10 years	2.84	17.18	(5.25–56.21)	<0.0001
≥90 vs. <10 years	2.35	10.46	(2.88–38.01)	0.0004
Drug counts	​	​	​	​
≥5 drugs vs. <5 drugs	1.14	3.11	(2.58–3.76)	<0.0001
Drug prescription days	​	​	​	​
30–59 days vs. <30 days	2.41	11.10	(7.07–17.44)	<0.0001
≥60 days vs. <30 days	3.36	28.89	(19.53–42.74)	<0.0001
Prescribing departments counts	​	​	​	​
2 departments vs. 1 department	0.37	1.44	(1.18–1.76)	0.0004
≥3 departments vs. 1 department	0.74	2.09	(1.59–2.76)	<0.0001
Interaction terms (variables × age group)				
Sex × age group	—	—	—	0.554
Drug counts × age group	—	—	—	0.016
Drug prescription days × age group	—	—	—	<0.0001
Prescribing departments counts × age group	—	—	—	0.002

The results are derived from the global model using leftover drug status as the dependent variable, with all clinical factors treated as categorical variables. Adjusted odds ratios (AOR), 95% confidence intervals (95% CI), and partial regression coefficients (*β*) are presented. The *p*-values for the interaction terms between each factor and age group are shown at the bottom of the table. The model’s performance was assessed using generalized *R* ^*2*^ (0323) and area under the curve (AUC = 0.858).

#### Stratified and sensitivity analyses

**FIGURE 3:**
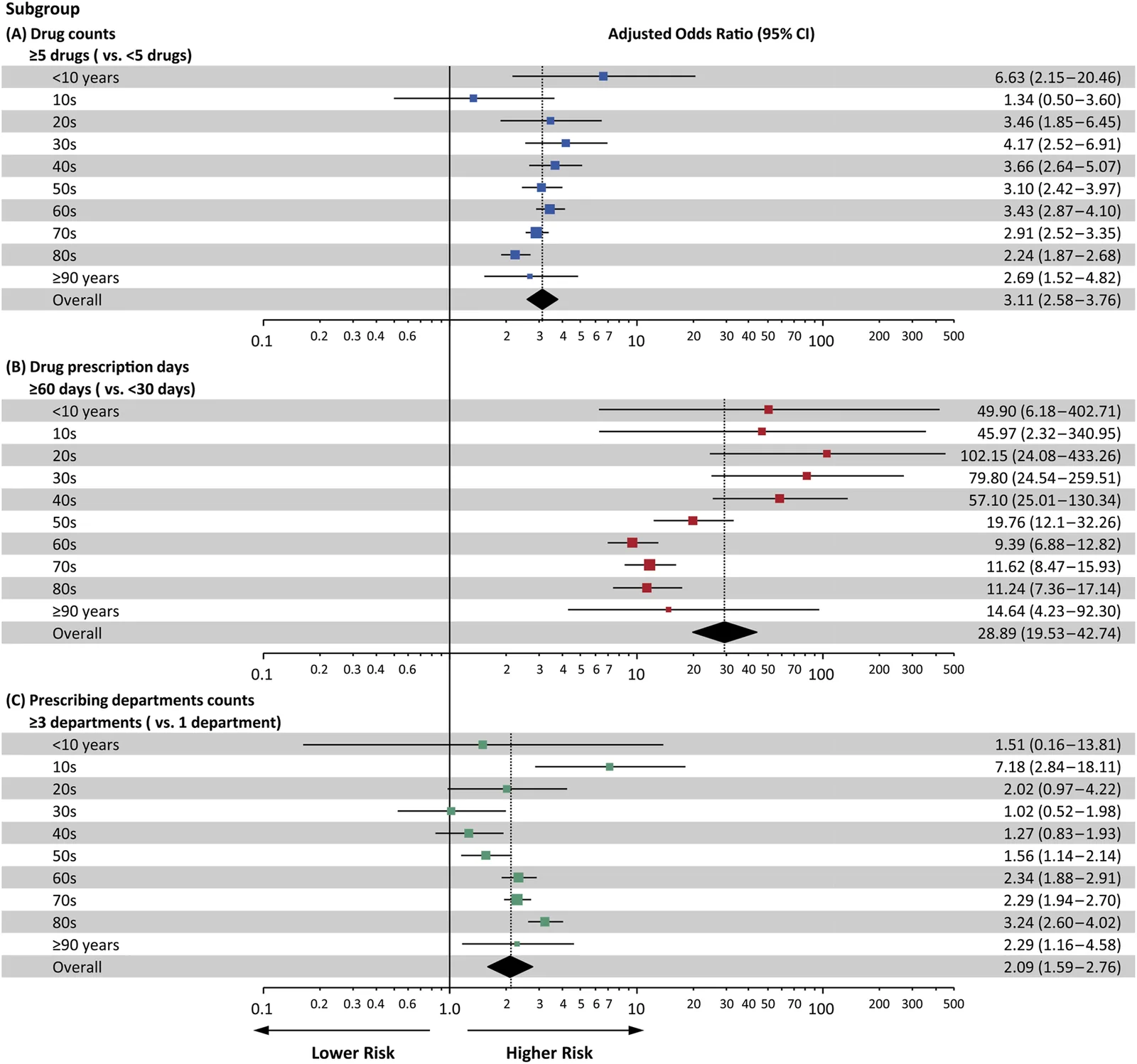
Age-specific adjusted odds ratios for factors associated with leftover drugs. The forest plot presents age-stratified adjusted odds ratios (AORs) for the three primary factors, exhibiting significant interactions with age in relation to the presence of leftover drugs. The analysis compares high-risk categories to their respective reference groups: **(A)** drug counts ≥5 versus <5, **(B)** prescription days ≥60 versus <30, and **(C)** prescribing departments ≥3 versus 1. To facilitate a direct comparison of the relative impact of these clinical factors, all sections utilized a unified logarithmic scale ranging from 0.1 to 500. The colored squares represent the AOR for each age group (marker size ∝ sample size) and solid black diamonds indicate the overall AOR for the entire cohort. Horizontal lines denote the 95% confidence intervals (CIs), with corresponding numerical values provided on the right for precise reference. The vertical solid line at AOR = 1.0 represents the null effect and the labels at the bottom indicate the direction of risk. Points to the right of the vertical line (AOR >1.0) signify a higher risk of leftover drugs than in the reference group. Complete results, including the intermediate categories, are shown in Supplementary Table S2.

Age-stratified logistic regression was performed for factors with significant interactions identified in the global model to evaluate their effects within each age category. Factors showing significant interactions in the global model were included as covariates in these stratified models, and the AORs and 95% CIs were compared across age groups. The results of the age-stratified analyses, including the primary risk categories, were visualized using a forest plot (Figure 3). To ensure the clarity of the figure, the AORs for the highest-risk categories—drug counts ≥5, prescription days ≥60, and prescribing departments ≥3—are prioritized for presentation (Figure 3), while the comprehensive results of the stratified analyses for all categories are provided in the Supplementary Table S2. Furthermore, sensitivity analysis was conducted to verify the robustness of the AOR estimates for the primary factors. Specifically, all the clinical factors under consideration were re-entered as covariates into the stratified models, regardless of the significance of their interactions in the global model, to evaluate the impact of their inclusion on the AORs of the primary factors.

#### Predictive probability plots

**FIGURE 4:**
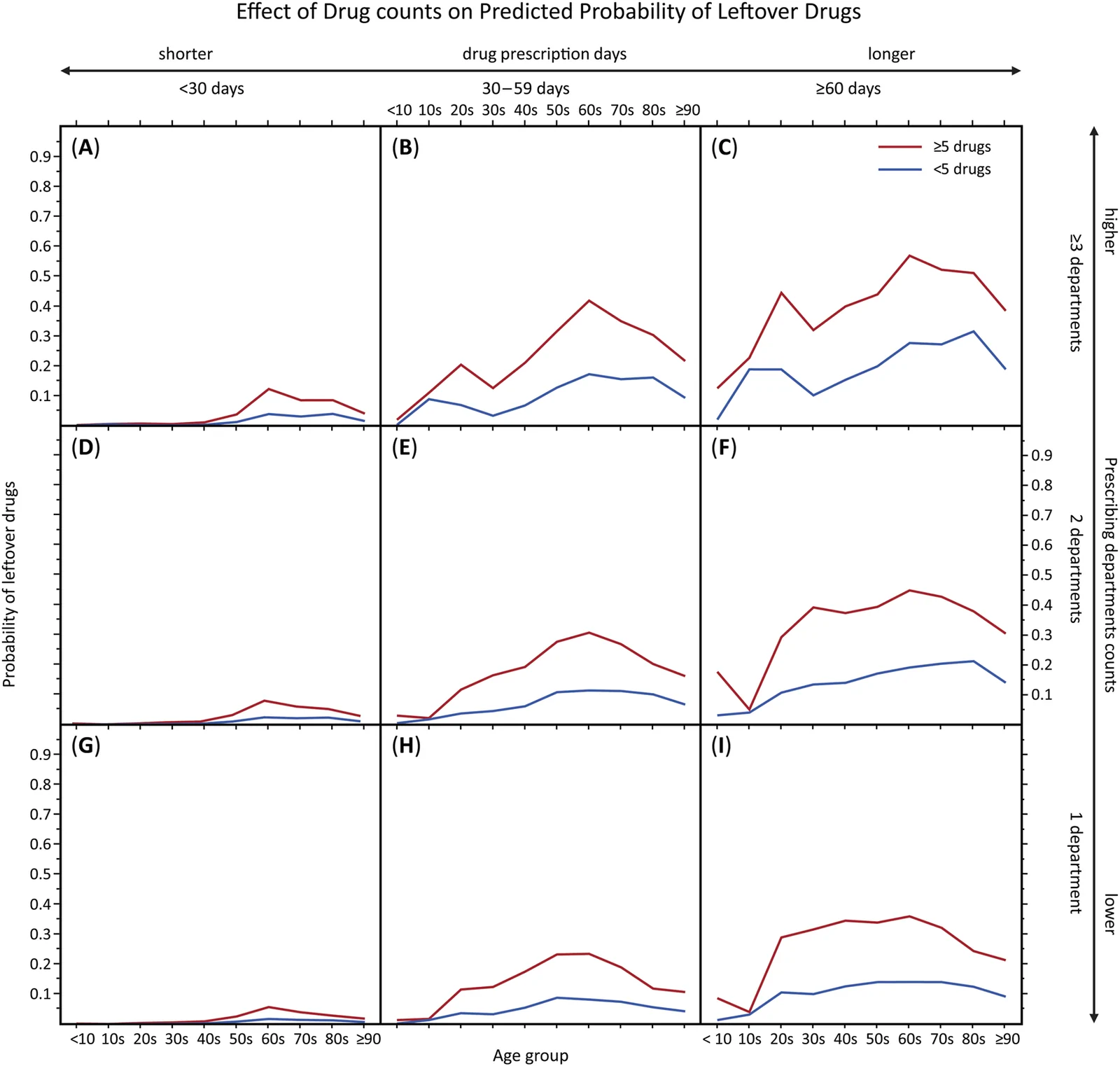
Predicted probability of leftover drugs by age group and drug counts. Based on the multivariate model including interaction terms, this plot shows the age-dependent impact of drug counts (<5 vs. ≥5 drugs) on the probability of leftover drug occurrence. The nine-panel matrix **(A–I)** comprehensively illustrates these probabilities across all the combinations of prescription days and prescription department counts. Predicted probabilities (*P*) were calculated using the logistic function *P* = 1/ (1 + exp(−(*β*_0_ + ∑*β*_i_ *X*_i_))), where *β* represents the regression coefficient for each factor. Model performance was evaluated using the generalized *R* ^2^ (0.323) and area under the curve (AUC = 0.858).

**FIGURE 5:**
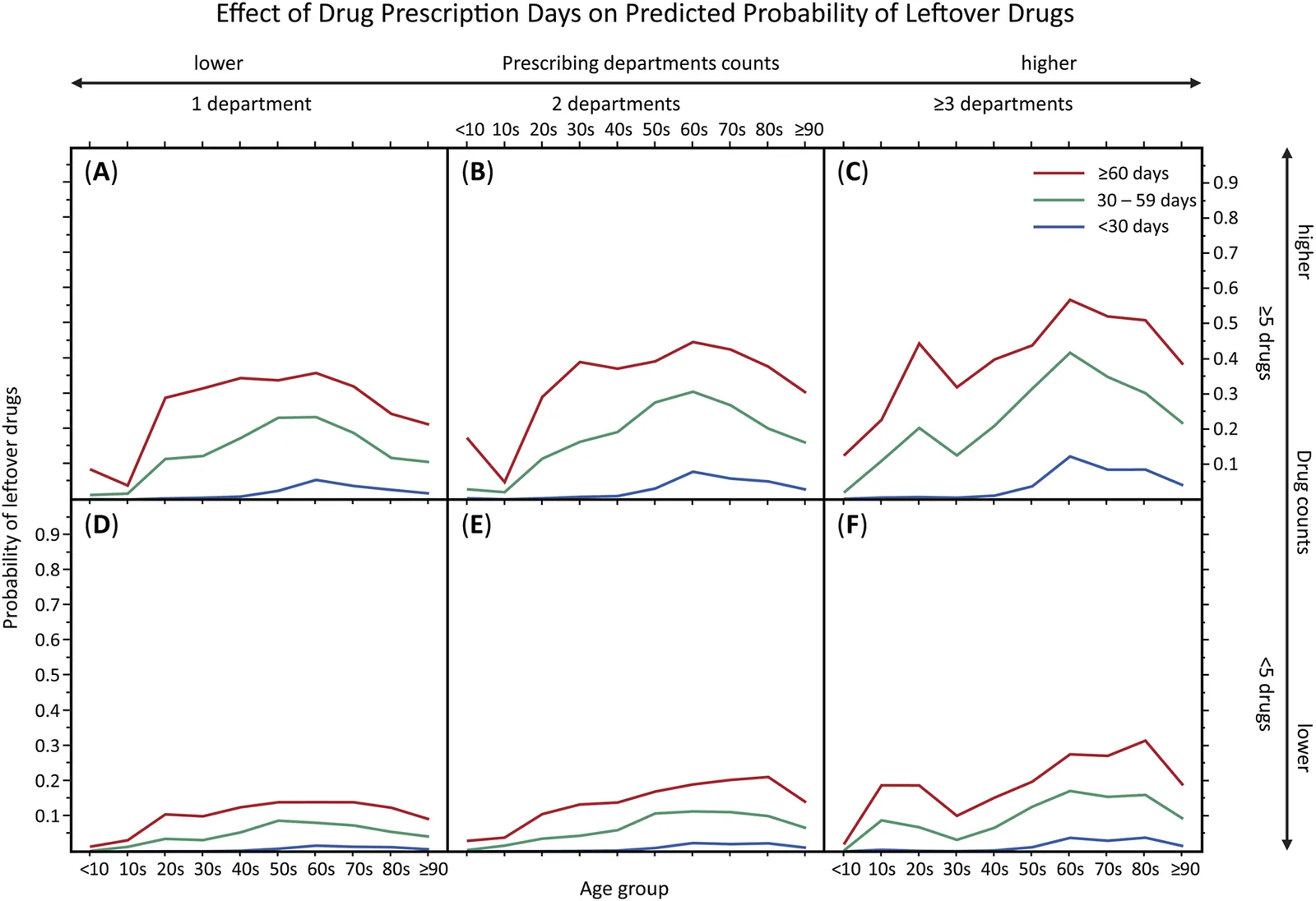
Predicted probability of leftover drugs by age group and drug prescription days. The figure shows the age-dependent changes in the impact of drug prescription days (<30, 30–59, and ≥60 days) on the leftover drug risk. The six-panel matrix **(A–F)** highlights markedly increased risks associated with long-term prescriptions, particularly in younger age groups, adjusted for other clinical factors. Predicted probabilities (*P*) were calculated using the logistic function *P* = 1 / (1 + exp(−(*β*_0_ + ∑*β*_i_ *X*_i_))), where *β* represents the regression coefficient for each factor. Model performance was evaluated using the generalized *R* ^2^ (0.323) and area under the curve (AUC = 0.858).

**FIGURE 6:**
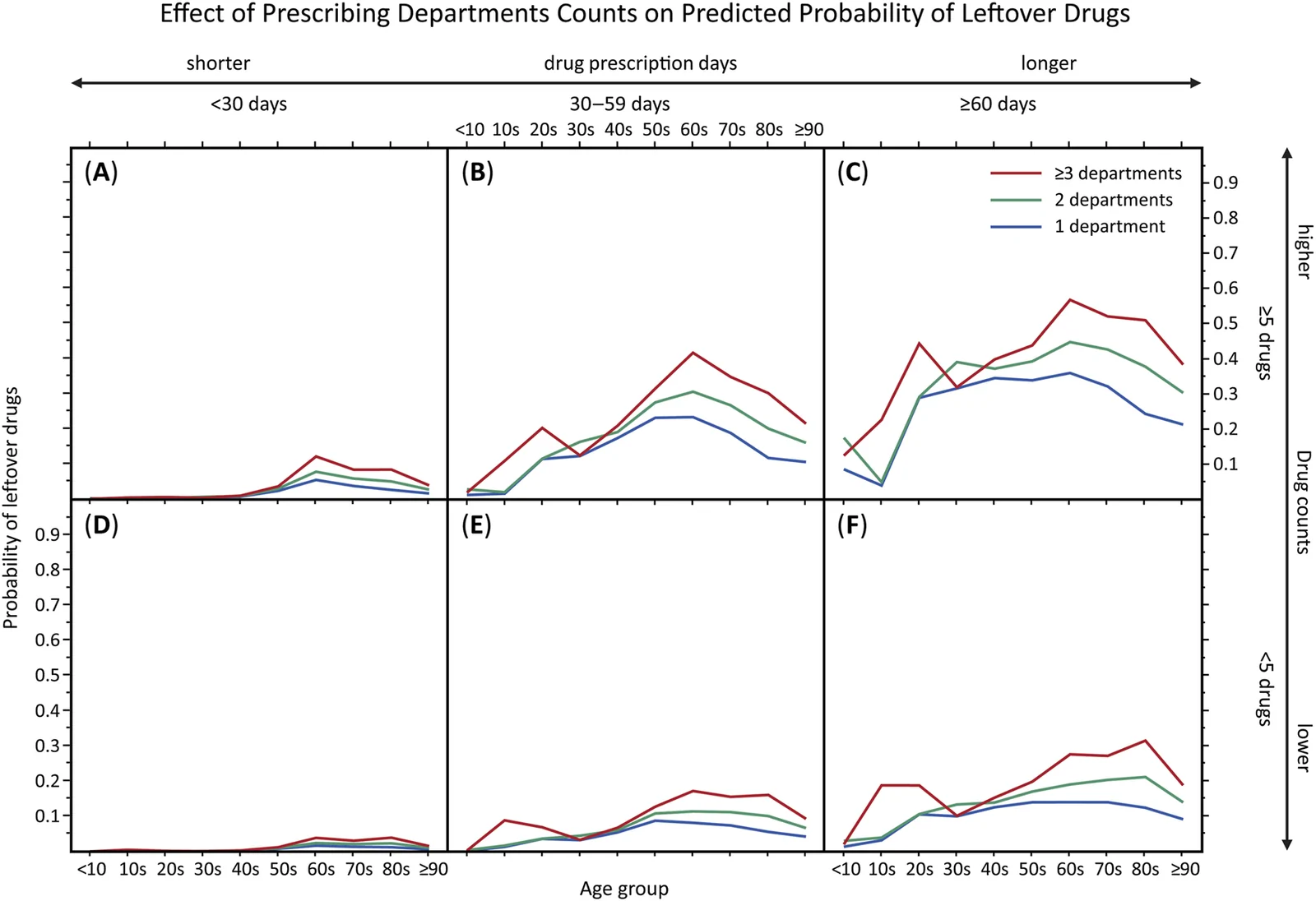
Predicted probability of leftover drugs by age group and prescribing departments’ counts. The age-dependent influence of the number of prescribing departments (1, 2, or ≥3) on the predicted probability of leftover drugs is plotted. The six-panel matrix **(A–F)** provides an overview of the evolution of this risk across different drug counts and prescription day profiles. Predicted probabilities (*P*) were calculated using the logistic function *P* = 1 / (1 + exp(−(*β*_0_ + ∑*β*_i_ *X*_i_))), where *β* represents the regression coefficient for each factor. Model performance was evaluated using the generalized *R* ^2^ (0.323) and area under the curve (AUC = 0.858).

A series of predicted probability plots (Figures 4–6) was generated based on the results of the multivariate logistic regression model, including interaction terms, to visually clarify the age-dependent changes in the association between primary factors and the occurrence of leftover drugs. The predicted probability (*P*) of the leftover drug occurrence was calculated using the following logistic function:

$$P=\frac{1}{\left(1+\exp\;\left(-\left(\;\beta_{0}+\beta_{1}X_{1}+\beta_{2}X_{2}+...+\beta_{k}X_{k}\right)\right)\right)}$$

where, *β*_0_ represents the intercept, *β*_i_ represents the regression coefficients for each independent variable estimated from the model, and *X*_i_ represents the level values of each factor. In this analysis, the predicted probabilities were comprehensively calculated for all 360 possible combinations of levels across sex, age, department count, drug count, and prescription days. By analyzing all profiles rather than fixing specific covariates to reference categories, we comprehensively visualized the complex interactions between the factors for each primary factor. These figures present the overall predicted trends after adjusting for the covariate effects.

For each primary factor of interest, a matrix-like panel configuration was used (Figures 4A–I, 5A–F and 6A–F), where the levels of the remaining two factors corresponded to rows and columns. In each panel, the vertical axis (y-axis) represents the predicted probability (*P*, 0–1.0), and the horizontal axis (x-axis) represents the age group. Within the panels, the predicted curves corresponding to each factor level are shown on the same graph. Only point estimates for each predicted curve are shown to present the overall trends derived from the multivariate model concisely and clearly.

## Results

### Characteristics of the study population

From the initial pool of 42,122 outpatients who visited our hospital and received medical prescriptions between April 1, 2016, and March 31, 2021, 36,340 patients were included in the final analysis (Figure 1). Based on the leftover drug adjustment protocol, 3,472 patients (9.6%) had leftover drugs and were categorized into the drug leftover (+) group (Figure 1; Table 1). The overall proportion of leftover drugs was 9.6%. Among the 3,472 patients in the Drugs leftover (+) group, single occurrences accounted for 46.3% (*n* = 1,608), while multiple occurrences (≥2 times) accounted for 53.7% of the cases (Supplementary Table S1). When stratified by age group, single occurrences were most prominent in the younger age groups (e.g., 69.4% in the 10s and 55.2% in the 20s). Conversely, the proportion of patients with multiple reports (≥2 times) increased with age, accounting for more than half of the cases in middle-aged and older populations (≥40 years).

In the comparison of baseline clinical characteristics (Table 1), significant differences were observed for all factors, except sex (*p* = 0.261). Compared with those in the Drugs leftover (−) group (*n* = 32,868), individuals in the Drugs leftover (+) group were significantly older (mean 67.9 vs. 51.3 years) and exhibited significantly higher mean values for drug counts (4.5 vs. 2.3 drugs), prescription duration (75.6 vs. 38.1 days), and the number of prescribing departments (2.1 vs. 1.4) (all *p* < 0.0001).

### Preliminary and age-stratified analysis

The observed prevalence of leftover drugs by age group showed a stepwise upward trend with age, increasing from 0.4% in those under 10 years to 15.9% in the 80s group, before decreasing to 10.5% in those aged ≥90 years (Figure 2). Age-stratified comparisons of clinical factors (Table 2) revealed that across nearly all age groups, including the ≥90 years cohort, the Drugs leftover (+) group exhibited significantly higher values for drug counts, prescription duration, and prescribing department counts compared with the Drugs leftover (−) group (*p* < 0.05). However, in the under 10 years group, the mean drug count did not show a statistically significant difference between the two groups (*p* = 0.263), although the proportion of patients with ≥5 drugs remained significant (*p* < 0.0001).

Regarding sex, no significant difference was observed between the two groups in most age categories, including the <10 years (*p* = 0.587) and ≥90 years (*p* = 0.668) cohorts. In contrast, significant differences were identified, particularly in the 70s (*p* = 0.030) and 80s (*p* = 0.018) age groups.

### Multivariate analysis and interaction testing

The global multivariate logistic regression model demonstrated high performance with an AUC of 0.858 and a generalized *R* ^2^ of 0.323 (Table 3). Age group and all primary factors showed significant interactions, except sex (*p* = 0.554), drug counts (*p* = 0.016), drug prescription days (*p* < 0.0001), and prescribing department counts (*p* = 0.002) (Table 3).

### Age-specific impact of primary factors

AOR and predicted probabilities were analyzed for factors with significant interactions. To ensure clarity of the figure, the AORs for the highest-risk categories of each factor are prioritized and shown in Figure 3. Comprehensive results across all categories are provided in the Supplementary Table S2.

Regarding drug counts, the AOR for polypharmacy (≥5 drugs vs. <5 drugs) was 3.46 (95% CI: 1.85–6.45) in the 20s and 4.17 (95% CI: 2.52–6.91) in the 30s, demonstrating a downward trend with age (Figure 3A; Supplementary Table S2). Figure 4 indicates that predicted probabilities for ≥5 drugs (red line) consistently exceeded those for <5 drugs (blue line) across all profiles. Furthermore, older patients in their 60s–80s maintained a higher baseline probability, even with fewer than five drugs, compared with their younger counterparts.

The impact of drug prescription days was notable, with a substantially high AOR for long-term prescriptions (≥60 days versus <30 days) in younger groups, reaching 102.15 (95% CI: 24.08–433.26) in the 20s and 79.80 (95% CI: 24.54–259.51) in the 30s (Figure 3B; Supplementary Table S2). On the unified logarithmic scale shown in Figure 3, this represented the largest risk increase observed across all age groups and clinical factors. Correspondingly, as shown in Figure 5, the predicted probability curve for ≥60 days (red line) exhibited a sharp upward slope in the 20s and 30s compared to other prescription durations.

For prescribing department counts, the AOR for ≥3 departments vs. 1 department was 7.18 (95% CI: 2.84–18.11) in the 10s and 3.24 (95% CI: 2.60–4.02) in the 80s (Figure 3C; Supplementary Table S2). Figure 6 illustrates a stepwise upward shift in the probability curves across age groups as the number of departments increased, highlighting the additive impact of multidepartment care.

### Combined effects of factors

Synergistic effects among clinical factors were identified through predicted probability analysis. Regarding the interaction between drug counts and prescription days, the probability increase associated with longer prescription durations was steeper in the group with ≥5 drugs (Figure 5, Panels A–C vs. D–F). Under the specific profile of ≥5 drugs and ≥60 prescription days, probabilities remained above 0.3 from the 30s to the 80s, eventually reaching approximately 0.6.

An additional risk related to the number of prescribed departments was observed. As shown in Figure 6, stepwise upward shifts were evident in each panel (A–F) as department counts increased, whereas drug counts and days remained fixed. For patients with <5 drugs and ≥3 departments (Figure 6F), predicted probabilities were approximately two- to three-times higher than for those receiving care from a single department.

A unique trend was identified in younger patients; under the condition of <5 drugs, ≥3 departments, and ≥60 prescription days (Figure 5F), the predicted probabilities for those in their 20s and 30s peaked above 0.2. In contrast, in profiles where all factors were at baseline levels, defined as <5 drugs, <30 days, and 1 department (Figures 4G, 5D, 6D), the predicted probabilities remained consistently below 0.05 across age groups.

## Discussion

The primary objective of this study was to elucidate age-specific factors associated with the occurrence of leftover drugs among outpatients by utilizing leftover drug reporting data based on the PBPM by community pharmacists. Detailed investigation revealed that the leftover drug groups accounted for 9.6% of the total population. Compared with the non-leftover group, this group comprised significantly older patients, with higher drug counts, longer prescription durations, and a greater number of prescribing departments. These findings align with those of previous studies, suggesting that medication adherence tends to decrease in older adults [11–13]. Although leftover drugs are often used as an indicator of potential adherence issues, they do not exclusively represent medication nonadherence. Multiple clinical circumstances, including dose adjustments, treatment discontinuation, hospitalization, and deprescribing, may contribute to leftover drugs. Therefore, the findings of this study should be interpreted in the context of leftover drugs as a multifactorial clinical indicator. Conversely, a certain proportion of leftover drugs was confirmed among younger age groups, suggesting that the issue of leftover drugs is a shared challenge across all generations, rather than being limited to older adults. The novelty of this study lies in the use of actual leftover drug adjustment data based on the PBPM to perform a decade-stratified analysis, thereby clarifying the interactions between age and each clinical factor (drug count, prescription days, and department count). This approach revealed that the factors contributing to the occurrence of leftover drugs differ dramatically between younger and older populations, highlighting the necessity for age-specific countermeasures.

Regarding sex, although significant differences were observed in the 70s (*p* = 0.030) and 80s (*p* = 0.018) groups in the univariate analysis (Table 2), it was not a significant independent factor or showed any interaction with age in the multivariate logistic regression analysis (Table 3). This suggests that the sex differences observed in specific age groups may stem from other clinical backgrounds, such as variations in drug counts or the number of prescribing departments. Previous studies have reported inconsistent findings regarding the effects of sex on medication adherence [14–16]. Our results emphasize that the fundamental drivers of leftover drug risk are “prescription complexities,” such as polypharmacy and prescription duration, rather than demographic attributes like sex. Therefore, in clinical settings, medication support may benefit from prioritizing interventions based on each patient’s prescription profile rather than distinctions based on sex.

Multivariate logistic regression analysis demonstrated significant interactions between age groups and all analyzed factors, except sex, indicating that the risk structure for leftover drug occurrence shifts substantially across life stages. While age, polypharmacy, and regimen complexity have previously been identified as important determinants of medication adherence [17–21], in most investigations, these factors were evaluated independently or within specific patient populations. In contrast, our lifespan-based approach allowed us to identify how the relative influence of prescription duration, drug count, and care fragmentation shifts across different stages of life. The significant interaction effects observed by us indicate that age is not merely an independent factor associated with leftover drugs but also modifies how prescription characteristics influence adherence-related challenges. Younger adults (<60 years) are generally reported to exhibit lower adherence than older adults [17, 18, 22]. Consistent with this, in our study, long-term prescription emerged as the most potent risk factor for younger adults, particularly those in their 20s–40s. In contrast, multi-department visits and polypharmacy were more strongly associated with leftover drugs in older patients. These findings demonstrate that the underlying factors contributing to leftover drugs are qualitatively distinct across different age groups. Consequently, uniform interventions are insufficient, and age-stratified approaches are essential for effective medication management.

Regarding the number of medications, the drug count in the leftover group was approximately twice that of the non-leftover group, indicating that polypharmacy is associated with an increased risk of leftover drugs. This trend was particularly prominent in the middle-aged patients. Polypharmacy increases the medication burden and results in a higher incidence of adverse events. It is also a primary factor in the decline of medication adherence among older adults [11, 12, 19, 23]. Therefore, optimizing multidrug prescriptions is crucial; however, it is often not easy to reduce the number of medications required for disease treatment other than by switching to fixed-dose combinations. In response, some reports have pointed out that dosing frequency or regimen complexity is more important than the absolute number of medications [20, 21, 24]. Nevertheless, the predicted probability analysis (Figure 4) revealed that having fewer than five prescribed drugs was consistently associated with a lower likelihood of leftover drugs, even in the presence of other risk factors such as long-term prescriptions or multi-department visits. The strong association between polypharmacy and leftover drugs observed in older adults is in agreement with previous studies in which increasing medication burden was observed to contribute to poorer adherence and greater treatment complexity [14, 18, 19]. Notably, our predicted probability analysis further indicated that the detrimental effects of polypharmacy were amplified when combined with long prescription durations or care from multiple departments. This finding supports the growing recognition that medication-related risks in older adults arise not from a single factor but from the cumulative burden of multiple interacting components of pharmacotherapy. As a countermeasure, optimizing medication regimens through deprescribing [25], guided by tools, such as the Beers Criteria [26], STOPP/START criteria [27], and Guidelines for Medical Treatment and Safety in the Elderly in Japan [28], is considered highly effective.

Regarding prescription duration, the findings of this study provide a new perspective on conventional clinical assumptions. Long-term prescriptions are generally intended to reduce patient burden and enhance convenience [29]. However, our results showed that, among young adults, long-term prescriptions were strongly associated with the presence of leftover medication. In particular, among individuals in their 20s, the adjusted odds ratio (AOR) for prescriptions lasting 60 days or longer reached 102.15. Although the mechanisms underlying this association were not examined in the present study and remain to be clarified in future research, as shown in Supplementary Table S1, the predominance of single leftover drug occurrences in younger groups indicates that the non-adherence in these groups tends to be episodic (non-recurrent). This pattern aligns well with the hypothesis that, in younger adults, leftover drugs are primarily driven by situation-dependent behaviors, rather than a chronic inability to manage medications [17, 22]. Therefore, for younger patients, implementing individualized interventions that begin with short-term prescriptions to assess their management capabilities, combined with refilling prescriptions and pharmacist follow-up utilizing ICT, may be an effective approach. In contrast, for older adults, because leftover drugs are strongly associated with multidepartment visits and polypharmacy, simplifying the number of prescribed medications is a critical countermeasure. The increased proportion of recurrent leftover drug reports (≥2 times) in older populations (Supplementary Table S1) further suggests the chronic nature of medication management failure caused by such structural complexities. Furthermore, the lack of medical coordination may influence multidepartmental visits, underscoring the need to establish information-sharing systems between medical institutions in the future. In summary, the key elements of leftover drug countermeasures are “management of prescription duration” for younger patients and “medical coordination and simplification of drug counts” for older patients. Additionally, the use of split or refill prescriptions is beneficial because it enables continuous follow-up by pharmacists, while maintaining patient convenience. Furthermore, pharmacists in Japan are legally obligated to monitor the medication status throughout the treatment period [30]. As part of this obligation, follow-up on medication status after dispensing is an effective strategy for addressing the issue of leftover drugs.

Regarding the number of prescribing departments, visits to three or more departments were associated with an increased risk of leftover drugs. While it is generally reported that drug counts increase alongside the number of comorbidities in older patients [31], the risk of “medical fragmentation,” in which a lack of information sharing between departments leads to duplicate prescriptions or inappropriate drug additions, is a challenge shared across all age groups. Although some reports indicate a trend toward rising non-adherence rates among the oldest-old (aged 80 years and older) [18], the decrease in the leftover drug rate observed in our 90-year-old and older group (Figure 2) may be influenced by a transition to medication management by family members or caregivers. This suggests that an analysis from a perspective different from simple changes in individual self-management capabilities is necessary. When information sharing among clinical departments is insufficient, duplicate or unnecessary prescriptions may occur. Therefore, a system for centralized management of medication information is necessary. This should include the utilization of electronic health records for coordinating information between clinical departments within hospitals, and leveraging electronic prescriptions for coordinating between medical institutions and pharmacies.

The predicted probability analysis (Figures 4–6) revealed a synergistic risk amplification structure associated with the overlap of multiple factors, an insight obscured when individual factors are examined in isolation.

In younger and prime-age groups (10s–30s), while the risk of leftover drugs remains at an extremely low level under normal conditions, a certain “fragility” was observed, characterized by a non-linear surge in risk when specific factors coincide. Notably, even when the drug count was fewer than five, the combination of multi-department visits (≥3 departments) and long-term prescriptions (≥60 days) was associated with the predicted probabilities in the 20s and 30s peaking above 0.2 (Figure 5F). This suggests that even among younger individuals who are generally regarded as having high self-management capabilities, adherence tends to decline in the presence of lifestyle- or medication-related barriers and the complexity of pharmacotherapy [17, 22], indicating the existence of a “threshold” at which the convergence of complex consultation patterns and long-term management burdens may make it difficult to maintain adherence.

In the middle-aged group (40s–50s), the sensitivity of risk to an increase in the number of drugs was more prominent than that in the other age groups. Individuals in this life stage often have busy lifestyles because of employment and other social responsibilities. The introduction of polypharmacy (≥5 drugs) may make this cohort the most vulnerable to the negative impacts of extended prescription durations. In this group, suppressing the drug count to fewer than five may serve as an effective strategy for balancing work and treatment while potentially reducing the risk of leftover drugs.

In pharmacotherapy for older adults, various factors—including cognitive function, health beliefs, polypharmacy, and potentially inappropriate prescribing—influence treatment adherence and interact with one another [18, 19]. In this study, for the older population (aged 60 years and above), a structure became evident in which the risk was synergistically “amplified” based on a complex dependency wherein the number of medications, prescription days, and department counts complexly interacted with one another.

The interaction between polypharmacy (≥5 drugs) and other factors was notable. Under conditions with a high drug count, extended prescription durations were associated with a non-linear surge in the leftover drug risk, and the addition of multi-department visits was associated with a further increase in risk, reaching a maximum predicted probability of approximately 0.6 (Figures 5, 6). This suggests that a high volume of medication may increase vulnerability to other adverse conditions, such as difficulties in long-term management and information fragmentation across medical departments [31].

The most clinically significant finding observed consistently across age groups was the low risk associated with maintaining a drug count of fewer than five. The data revealed that even when prescription duration or the number of departments was unfavorable (e.g., ≥60 days or ≥3 departments), the probability of leftover drugs remained at a consistently low level as long as the drug count was appropriately controlled. These findings demonstrate important associations between drug count and occurrence of leftover drugs and suggest that drug count may be centrally associated with leftover drug occurrence across age groups.

Consequently, while simultaneous management of multiple factors may be desirable for potentially reducing the risk of leftover drugs, a key strategic priority may be to optimize the number of medications (deprescribing) across generations; this principle appears applicable regardless of age [25]. Combined with this, an age-specific tailored intervention approach, avoiding complex consultations and long-term prescriptions for younger adults and focusing on consolidating medical departments and inter-institutional coordination for older adults, may serve as an effective countermeasure against leftover drugs.

The primary strength of this study lies in the use of highly objective leftover drug adjustment data based on PBPM in community pharmacies. Unlike conventional adherence evaluations based on self-reported surveys, this study was based on the “clinical facts” of adjustments made through actual pharmacist intervention and consensus with prescribing physicians, enabling an analysis that more closely reflects real-world clinical practice. Furthermore, the statistical evidence demonstrating that the risk structure of leftover drug occurrence shifts substantially across life stages, achieved through detailed decade-stratified analysis and verification of interactions using multivariate logistic regression models, complements the existing knowledge. The visualization of synergistic risk amplification when multiple clinical factors overlap, facilitated by predicted probability plots, provides practical guidance for identifying and prioritizing high-risk groups in clinical settings.

This study had certain limitations, which should be considered when interpreting the results. First, there are constraints related to study design and selection bias. This was a retrospective investigation conducted at a single center and included only patients who disclosed leftover drugs to pharmacists under the PBPM protocol. Consequently, it did not account for patients who concealed their leftover drugs, those who discarded them at home before visiting the pharmacy, or cases in which prescription adjustments were already made during the medical examination at the clinic. Furthermore, as this was a single-center study, details regarding prescriptions and consultation behaviors outside the target facility could not be fully captured, and the possibility that regional characteristics or specific institutional prescription habits influenced the results cannot be ruled out.

The second limitation is the influence of unmeasured confounders. Although sex, age, drug count, prescription days, and prescribing department count were included in the model, psychosocial factors that are significantly associated with adherence were not considered. These include the patient’s socioeconomic status (financial burden and living alone), cognitive function, disease severity, health literacy, and trust in the relationship between the patient and healthcare providers. In particular, the remarkably high odds ratios observed in the younger age groups may be associated with behavioral factors, such as irregular lifestyles or intentional medication non-adherence, reported in previous studies [17, 22]. However, these factors were not directly measured in the present study, and therefore this interpretation should be considered hypothesis-generating rather than causal. Moreover, detailed subgroup analyses were not performed to assess the impact of factors other than the included covariates, such as whether specific drug classes (e.g., psychotropic drugs or self-adjusted medications) influenced the interactions, and it cannot be denied that these may have inflated the risk in specific age groups. Furthermore, an important limitation of this study is that leftover drugs do not necessarily reflect medication nonadherence alone. They may also arise from clinically appropriate circumstances, including physician-directed dose adjustments, treatment discontinuation, hospitalization, completion of therapy, or deprescribing. Therefore, leftover drugs should be interpreted as a multifactorial indicator and a proxy measure of potential adherence-related challenges rather than a direct measure of medication nonadherence.

The third limitation is related to the analytical methods and the temporal nature of the data. To ensure clinical interpretability and to accurately capture non-linear shifts in risk factors across life stages, age was analyzed as a categorical variable rather than a continuous one. Although the robustness of the primary factors was confirmed through sensitivity analysis, age was defined based on the “first visit” during the study period. Therefore, the dynamics of patients who transitioned across age groups during the five-year study period were not considered. Additionally, although data on the frequency of leftover drug reports were extracted and are summarized in Supplementary Table S1, we analyzed the primary outcome as a binary measure (presence [≥1 occurrence] versus absence [0 occurrences]). We opted for this approach because of the lack of established clinical criteria for categorizing leftover drug frequency and to maintain model clarity. While this binary approach helps identify objective cases of leftover drugs requiring actual prescription adjustments, it cannot quantify the exact volume of leftover medications. Furthermore, as shown in Supplementary Table S1, the data include both episodic occurrences (single intervention) and recurrent accumulations (recurrent interventions), which our multivariate analysis could not distinguish. Moreover, changes in adherence over time (longitudinal changes) were not evaluated, which limits the findings to a one-time, cross-sectional risk assessment.

The fourth limitation is regarding generalizability. The substantial increase in the risk associated with long-term prescriptions in younger groups (AOR 102.15) observed in this study may be heavily dependent on the specific context of the Japanese healthcare system and PBPM implementation. Thus, further verification is necessary to determine whether similar results can be obtained in different healthcare systems or with varying pharmacy practice models in other countries. Furthermore, as reflected by the exceptionally wide confidence intervals (e.g., 95% CI: 24.08–433.26 for prescriptions ≥60 days in patients in their 20s), the precise magnitude of these extreme odds ratios in younger populations entails substantial statistical uncertainty. This imprecision is primarily attributable to the sparse outcome events (e.g., only 14 patients in the <10-year group and 36 patients in the 10s group) and the extremely low baseline prevalence of leftover drugs within these younger age strata. Therefore, caution must be exercised while interpreting the magnitude of the observed effects in these subgroups. In the future, combining large-scale multicenter prospective studies with qualitative research capturing patient behavioral changes will enable the design of more comprehensive and precise countermeasures against leftover drugs.

## Conclusion

This study elucidated the age-specific characteristics of factors contributing to leftover drugs by utilizing objective reporting data based on PBPM by pharmacists. While advanced age, long-term prescriptions, multi-department visits, and polypharmacy were identified as the primary associated factors, the analysis revealed significant age-dependent interactions within the risk structure.

The paramount finding of this study was that long-term prescription represents an overwhelming risk factor, with an AOR exceeding 100 in younger adults (20s–40s), indicating a risk profile that is qualitatively distinct from that in older adults. Conversely, in older adults, polypharmacy and multidepartment visits interacted synergistically, further amplifying the risk of leftover drugs. Having fewer than five prescribed drugs was consistently associated with a lower likelihood of leftover drugs across age groups. However, prospective studies are needed to determine whether reducing medication burden itself decreases leftover medications or merely offsets the effects of other risk factors, such as longer prescription duration and a greater number of prescribing departments.

These findings suggest that the design of effective countermeasures necessitates an “age-specific tailored intervention” approach. This strategy should prioritize minimizing drug counts at its core, while simultaneously optimizing prescription duration according to the management capabilities of younger patients and strengthening prescription simplification and inter-institutional coordination for older adults. Such strategic interventions can contribute significantly to the realization of individualized pharmacotherapy that balances improved medication adherence with enhanced safety, economic efficiency, and optimal utilization of medical resources.

## Data Availability

The datasets used and analyzed in this study contain data related to patient information and therefore cannot be publicly shared due to institutional policies and the need to protect patient confidentiality. Aggregated data supporting the findings of this study are available from the corresponding author upon reasonable request and subject to the approval of relevant institutional authorities. Requests to access the datasets should be directed to TH, toshiyuki.hirai.dq@hitachi.com.
