# Supplementary material for: Age-related risk factors for leftover drugs: a lifespan analysis in community pharmacies
Source: J Pharm Pharm Sci. 2026 Sep 4;29:16879. doi: 10.3389/jpps.2026.16879 (PMC13586776; doi:10.3389/jpps.2026.16879)
Supplement: Supplementary file 1 [file Table1.docx]

**Supplemental Table 1.** Distribution of leftover drug report frequencies among patients with leftover drugs, stratified by age group.

|  | Overall |  | 1 occurrence | | 2 occurrences | | ≥3 occurrences | |
| --- | --- | --- | --- | --- | --- | --- | --- | --- |
|  | *n* = 3472 |  | *n* = 1608 | | *n* = 638 | | *n* = 1228 | |
| Age | *n* |  | *n* | Ratio (%) | *n* | Ratio (%) | *n* | Ratio (%) |
| Overall | 3472 |  | 1608 | 46.3% | 638 | 18.4% | 1228 | 35.3% |
| <10 years | 14 |  | 10 | 71.4% | 0 | 0% | 4 | 28.6% |
| 10s | 36 |  | 25 | 69.4% | 6 | 16.7% | 5 | 13.9% |
| 20s | 58 |  | 32 | 55.2% | 12 | 20.7% | 14 | 24.1% |
| 30s | 86 |  | 43 | 50% | 18 | 20.9% | 25 | 29.1% |
| 40s | 220 |  | 98 | 44.5% | 36 | 16.4% | 86 | 39.1% |
| 50s | 380 |  | 168 | 44.2% | 76 | 20% | 136 | 35.8% |
| 60s | 754 |  | 326 | 43.2% | 128 | 17% | 300 | 39.8% |
| 70s | 1174 |  | 547 | 46.6% | 228 | 19.4% | 399 | 34.0% |
| 80s | 686 |  | 327 | 47.7% | 128 | 18.7% | 231 | 33.7% |
| ≥90 years | 64 |  | 32 | 50% | 6 | 9.4% | 26 | 40.6% |

A total of 3,472 patients in the leftover drug group (Drugs leftover (+)) were stratified into 10-year age increments. Comparisons were made for the frequency of leftover drug reports per patient (1 occurrence, 2 occurrences, or ≥3 occurrences) across age groups. Data are presented as numbers (*n*) and percentages (%). Statistical significance for the differences in the frequency distribution was evaluated using the *χ^2^* test (*p* = 0.01674).

**Supplemental Table 2.** Age-stratified multivariate logistic regression analysis of factors associated with leftover drugs.

| **<10 years** | *n* = 3325, *p* < .0001, *R^2^* = 0.31, AUC = 0.92 | | |
| --- | --- | --- | --- |
| Variables | AOR | 95% CI | *p*-value |
| Drug counts |  |  |  |
| ≥5 vs. <5 | 6.63 | (2.15–20.46) | 0.001 |
| Drug prescription days |  |  |  |
| 30–59 days vs. <30 days | 7.24 | (0.44–118.01) | 0.164 |
| ≥60 days vs. <30 days | 49.90 | (6.18–402.71) | 0.0002 |
| Prescribing departments counts |  |  |  |
| 2 departments vs. 1 department | 2.18 | (0.65–7.36) | 0.208 |
| ≥3 departments vs. 1 department | 1.51 | (0.16–13.81) | 0.718 |
| **10s** | *n* = 2325, *p* < .0001, *R^2^* = 0.2, AUC = 0.83 | | |
| Variables | AOR | Variables | AOR |
| Drug counts |  |  |  |
| ≥5 vs. <5 | 1.34 | (0.50–3.60) | 0.560 |
| Drug prescription days |  |  |  |
| 30–59 days vs. <30 days | 19.42 | (2.32–162.70) | 0.006 |
| ≥60 days vs. <30 days | 45.97 | (6.20–340.95) | 0.0002 |
| Prescribing departments counts |  |  |  |
| 2 departments vs. 1 department | 1.27 | (0.50–3.22) | 0.614 |
| ≥3 departments vs. 1 department | 7.18 | (2.84–18.11) | <.0001 |
| **20s** | *n* = 2159, *p* < .0001, *R^2^* = 0.39, AUC = 0.92 | | |
| Variables | AOR | Variables | AOR |
| Drug counts |  |  |  |
| ≥5 vs. <5 | 3.46 | (1.85–6.45) | <.0001 |
| Drug prescription days |  |  |  |
| 30–59 days vs. <30 days | 32.60 | (6.75–157.56) | <.0001 |
| ≥60 days vs. <30 days | 102.15 | (24.08–433.26) | <.0001 |
| Prescribing departments counts |  |  |  |
| 2 departments vs. 1 department | 1.04 | (0.47–2.31) | 0.917 |
| ≥3 departments vs. 1 department | 2.02 | (0.97–4.22) | 0.061 |
| **30s** | *n* = 2725, *p* < .0001, *R^2^* = 0.36, AUC = 0.92 | | |
| Variables | AOR | 95% CI | *p*-value |
| Drug counts |  |  |  |
| ≥5 vs. <5 | 4.17 | (2.52–6.91) | <.0001 |
| Drug prescription days |  |  |  |
| 30–59 days vs. <30 days | 24.48 | (6.82–87.93) | <.0001 |
| ≥60 days vs. <30 days | 79.80 | (24.54–259.51) | <.0001 |
| Prescribing departments counts |  |  |  |
| 2 departments vs. 1 department | 1.39 | (0.81–2.38) | 0.237 |
| ≥3 departments vs. 1 department | 1.02 | (0.52–1.98) | 0.955 |
| **40s** | *n* = 3761, *p* < .0001, *R^2^* = 0.26, AUC = 0.83 | | |
| Variables | AOR | Variables | AOR |
| Drug counts |  |  |  |
| ≥5 vs. <5 | 3.66 | (2.64–5.07) | <.0001 |
| Drug prescription days |  |  |  |
| 30–59 days vs. <30 days | 22.89 | (9.57–54.75) | <.0001 |
| ≥60 days vs. <30 days | 57.10 | (25.01–130.34) | <.0001 |
| Prescribing departments counts |  |  |  |
| 2 departments vs. 1 department | 1.12 | (0.79–1.6) | 0.516 |
| ≥3 departments vs. 1 department | 1.27 | (0.83–1.93) | 0.269 |
| **50s** | *n* = 3984, *p* < .0001, *R^2^* = 0.26, AUC = 0.83 | | |
| Variables | AOR | 95% CI | *p*-value |
| Drug counts |  |  |  |
| ≥5 vs. <5 | 3.10 | (2.42–3.97) | <.0001 |
| Drug prescription days |  |  |  |
| 30–59 days vs. <30 days | 11.64 | (6.83–19.83) | <.0001 |
| ≥60 days vs. <30 days | 19.76 | (12.1–32.26) | <.0001 |
| Prescribing departments counts |  |  |  |
| 2 departments vs. 1 department | 1.26 | (0.96–1.65) | 0.092 |
| ≥3 departments vs. 1 department | 1.56 | (1.14–2.14) | 0.005 |
| **60s** | *n* = 5515, *p* < .0001, *R^2^* = 0.25, AUC = 0.80 | | |
| Variables | AOR | 95% CI | *p*-value |
| Drug counts |  |  |  |
| ≥5 vs. <5 | 3.43 | (2.87–4.1) | <.0001 |
| Drug prescription days |  |  |  |
| 30–59 days vs. <30 days | 5.12 | (3.59–7.31) | <.0001 |
| ≥60 days vs. <30 days | 9.39 | (6.88–12.82) | <.0001 |
| Prescribing departments counts |  |  |  |
| 2 departments vs. 1 department | 1.45 | (1.19–1.76) | <.0001 |
| ≥3 departments vs. 1 department | 2.34 | (1.88–2.91) | <.0001 |
| **70s** | *n* = 7613, *p* < .0001, *R^2^* = 0.23, AUC = 0.78 | | |
| Variables | AOR | 95% CI | *p*-value |
| Drug counts |  |  |  |
| ≥5 vs. <5 | 2.91 | (2.52–3.35) | <.0001 |
| Drug prescription days |  |  |  |
| 30–59 days vs. <30 days | 5.73 | (4.05–8.12) | <.0001 |
| ≥60 days vs. <30 days | 11.62 | (8.47–15.93) | <.0001 |
| Prescribing departments counts |  |  |  |
| 2 departments vs. 1 department | 1.57 | (1.34–1.84) | <.0001 |
| ≥3 departments vs. 1 department | 2.29 | (1.94–2.7) | <.0001 |
| **80s** | *n* = 4324, *p* < .0001, *R^2^* = 0.24, AUC = 0.79 | | |
| Variables | AOR | 95% CI | *p*-value |
| Drug counts |  |  |  |
| ≥5 vs. <5 | 2.24 | (1.87–2.68) | <.0001 |
| Drug prescription days |  |  |  |
| 30–59 days vs. <30 days | 4.68 | (2.93–7.47) | <.0001 |
| ≥60 days vs. <30 days | 11.24 | (7.36–17.14) | <.0001 |
| Prescribing departments counts |  |  |  |
| 2 departments vs. 1 department | 1.89 | (1.51–2.35) | <.0001 |
| ≥3 departments vs. 1 department | 3.24 | (2.60–4.02) | <.0001 |
| **≥90 years** | *n* = 609, *p* < .0001, *R^2^* = 0.25, AUC = 0.82 | | |
| Variables | AOR | 95% CI | *p*-value |
| Drug counts |  |  |  |
| ≥5 vs. <5 | 2.69 | (1.52–4.82) | 0.001 |
| Drug prescription days |  |  |  |
| 30–59 days vs. <30 days | 6.50 | (1.64–43.27) | 0.006 |
| ≥60 days vs. <30 days | 14.64 | (4.23–92.3) | <.0001 |
| Prescribing departments counts |  |  |  |
| 2 departments vs. 1 department | 1.61 | (0.80–3.24) | 0.184 |
| ≥3 departments vs. 1 department | 2.29 | (1.16–4.58) | 0.018 |

Participants were stratified into 10-year age increments to identify generation-specific clinical factors contributing to the occurrence of leftover drugs. For each age group, multivariate logistic regression analysis was performed using leftover drug status as the dependent variable. Adjusted odds ratios (AOR), 95% confidence intervals (95% CI), and *p*-values are presented for drug counts, drug prescription days, and prescribing department counts. Model performance for each sub-analysis is indicated by the sample size (*n*), generalized *R^2^*, and area under the curve (AUC).
